# A Three-Stage Fusion Neural Network for Predicting the Risk of Root Fracture—A Pilot Study

**DOI:** 10.3390/bioengineering12050447

**Published:** 2025-04-24

**Authors:** Yung-Ming Kuo, Liang-Yin Kuo, Hsun-Yu Huang, Tsen-Yu Sung, Chun-Hung Yang, Wan-Ting Chang, Chien-Shun Lo

**Affiliations:** 1 Department of Electronic Engineering, National Formosa University, Yunlin 632301, Taiwan; ymkuo@gs.nfu.edu.tw (Y.-M.K.); csimsong1@gmail.com (T.-Y.S.); 2 Smart Machinery and Intelligent Manufacturing Research Center, National Formosa University, Yunlin 632301, Taiwan; g9330808@gmail.com; 3 Department of Multimedia Design, National Formosa University, Yunlin 632301, Taiwan; 4 Department of Stomatology, Ditmanson Medical Foundation Chia-Yi Christian Hospital, Chiayi 600566, Taiwan; cych07114@gmail.com; 5 Department of Electrical Engineering, National Formosa University, Yunlin 632301, Taiwan; eli.yang@nfu.edu.tw

**Keywords:** root fracture, root canal therapy, neural networks, deep learning

## Abstract

Predicting the risk of root fractures following root canal therapy requires diagnosis of the dental history and status of patients. However, dental history is a kind of categorical data type that is not easy to combine with numerical data to obtain good performance in deep learning. The accuracy of support vector machine (SVM) and artificial neural networks (ANNs) is 71.7% and 73.1%, respectively. In this study, a three-stage fusion neural network (TSFNN) is proposed to improve the multiple types of clinical data in the dental field based on ANNs. Clinical data were obtained from 145 teeth, comprising 97 fractured teeth and 48 nonfractured teeth. Each dataset contained 17 items, which were divided into 10 categorical items and 7 numerical items. TSFNN combines numerical and categorical NN with batch normalization and embedding layer techniques and can produce the accuracy of 82.1% and a 19.1% improvement in F1-score. It shows impressive performance in predicting the risk of root fracture. Furthermore, due to the limited amount of clinical data, it is believed that such a pilot study can effectively improve the results when the amount of clinical data is insufficient.

## 1. Introduction

According to the research, root fracture following root canal therapy has an incidence of about 30% [1]. Most of root fractures are complete or incomplete longitudinal fractures along the long axis of the tooth, vertical root fracture (VRF) is called [2]. Concerning the relationship between root canal therapy and VRF, Sedgley indicates that shear strength, toughness and compressive strength of teeth decreases about 3.5% after root canal therapy [3]. Reeh [4], in addition indicates the loss of tooth stiffness was 5% after the endodontic procedures. Barreto [5] found that apical pressure filling techniques are factors causing VRF. VRF occurred in 13.3% of cases by lateral compaction and 33.3% of cases by Tagger’s hybrid. Wilcox [6] indicates that root canal enlargement may also induce VRF. Saw [7] mentioned the influence of different obturation techniques on root strains could cause VRF. Many researchers propose a variety of possible causes of VRF after root canal therapy. However, it was hard to predict the risk of VRF by integrating these different causal factors until artificial intelligence (AI) became significantly advanced. Sanjeev B. Khanagar [8] reviewed 43 research papers from the dental field from the past to 2021. It showed the use of AI in dental research increases from one paper in 2008 to 20 in 2019. It includes detection and diagnosis of dental caries, proximal dental caries, VRF, apical lesions and so on [9,10,11,12,13,14,15,16,17,18,19,20].

Most cases of AI medical applications mention deep learning used in their study [21,22,23]. In most cases, deep learning (DL) is achieved by neural networks (NNs). These neural networks include artificial neural networks (ANNs) [10,11,12], convolutional neural networks [13,14,15,16,17,18], Bayesian neural networks (BNNs) [19] and probabilistic neural networks (PNNs) [20]. ANN is the kernel architecture in this study.

**Figure 1:**
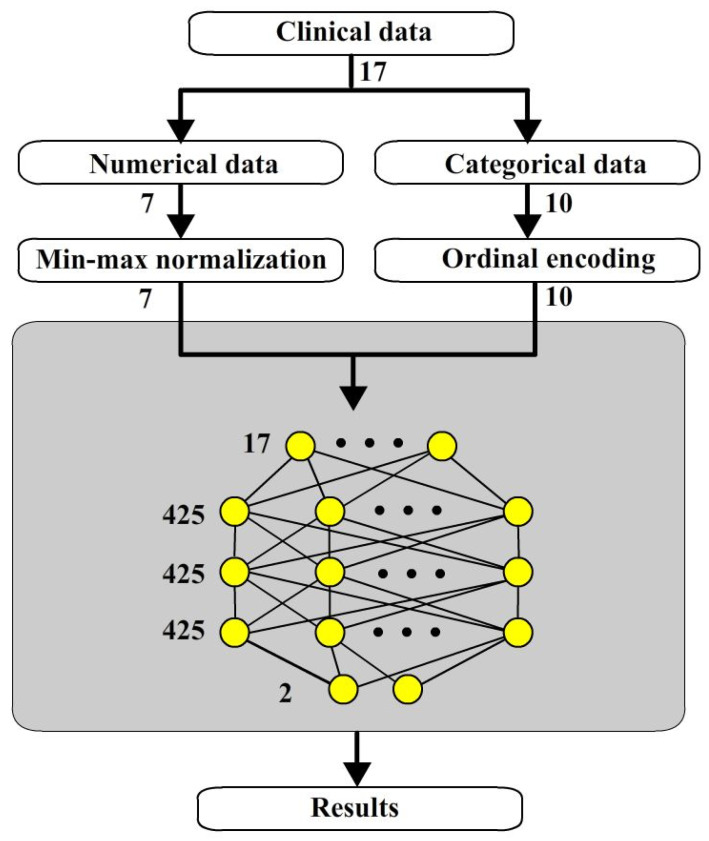
Original ANNs.

Compared to previous works, it was not until 2023 that Chang [12] integrated all practical factors to predict the risk of VRF by using deep learning neural network (DLNN). Utilizing the same variables used to predict VRF with these different approaches is proposed in this study. The data used in this study only contain 145 teeth with 17 variables. The data were collected over a period of six years in Taiwan. Taiwan has a population of approximately 23 million. Limited clinical data size is a common situation faced by medical research in Taiwan. In such situation, support vector machine (SVM) of machine learning (ML) may be similar to original ANNs. The original ANNs could be used as shown in Figure 1. The clinical data are classified into numerical and categorical data normalized separately and then input into to ANNs. In this way, the accuracy of ANNs is 73.1%, a little better than SVM which is 71.7%.

In this paper, a three-stage fusion neural network is proposed to improve the original ANNs. It could raise the accuracy from 73.1% to 82.1%. It does not only show a modification of approach but also of the architectures.

## 2. Materials and Methods

### 2.1. Data Collections

The clinical data used in this study were collected from January 2015 to June 2021 at the Department of Stomatology, Ditmanson Medical Foundation Chia-Yi Christian Hospital. In total, conforming clinical data were obtained from 145 teeth, comprising 97 fractured teeth and 48 nonfractured teeth.

### 2.2. Datasets

Each dataset contained 17 items, as shown in Table 1. These 17 items describe the dental history and status of patients. These 17 items were divided into numerical and categorical items. There are 10 categorical items and 7 numerical items.

#### 2.2.1. Categorical Data

As shown in Table 1, for example, items no 1 to 6 are questions of binary options. Items no. 7 to 10 are multiple options. These options do not accurately describe the measurable relationship between them. For example, sex includes male and female. There is no measurable relationship concerning teeth between male and female. The next example is that of no. 4, which records the preoperative pain. In this case, it is not possible to describe how much pain was experienced. As another example, there are a possible 6 positions of item no 7. However, there are no specific relationship between them in order. Items no. 1 to 10 are defined as categorical data.

#### 2.2.2. Numerical Data

In contrast, objects that can be measured are considered as numerical data. For example, the age at the time of treatment of case no 11 is a continuous and measured data. Remaining root canal wall thickness of case no 16 is a numerical data. In the same way, no 11 to 17 are considered as numerical data.

As a result, the options are numbers without comparable and continuous relationship between them could be considered as categorical data; in contrary, they are numerical data.

### 2.3. Stage 1 Numerical Neural Network (NNN)

#### 2.3.1. Architecture of Numerical Neural Network

**Figure 2:**
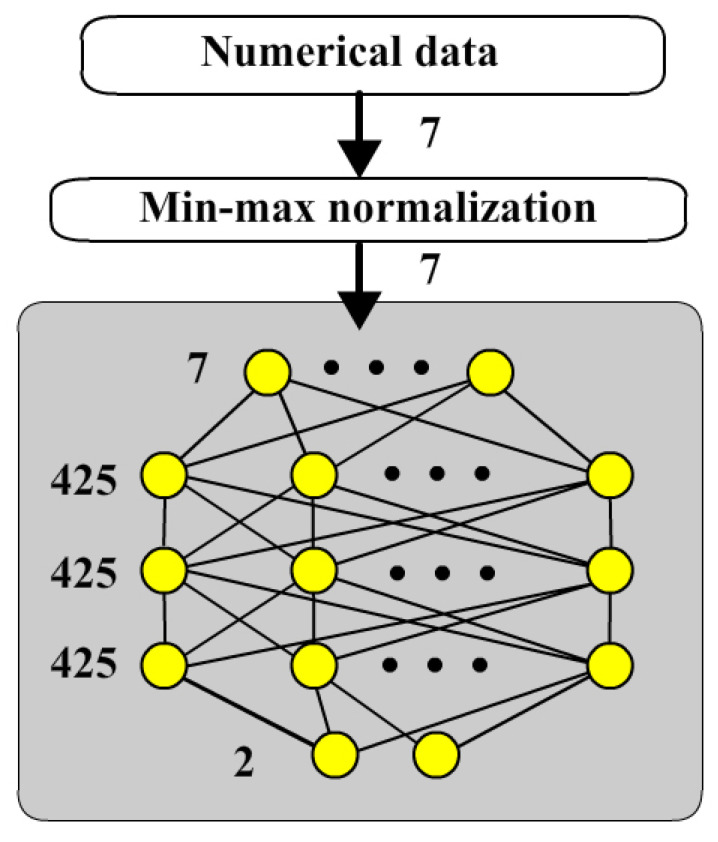
Numerical neural network.

The first stage is to separate the numerical data into min-max normalization and then into a neural network as shown in Figure 2. In our experiments, items no 11 to 17 were in parallel into min-max normalization model to transform data into a uniform range from 0 to 1.

#### 2.3.2. Min-Max Normalization for Numerical Items

Min-max normalization is used to map data into a uniform range to achieve the purpose of normalization. The output area is usually 0 to 1 or −1 to 1. Values 0 to 1 were used in our experiments. For each item (no 11 to 17), the minimal and maximal value of each item are used to map the specific item value to 0 to 1. The equation is shown as Equation (1)

y = x/(max(x) − min(x)),(1)

where x is the original value of items, max(x) and min(x) are the maximal and minimal value of each item. The normalized value y is obtained to as the input of DLNN for the 7 numerical items in our experiments.

### 2.4. Stage 2 Categorical Neural Network (CNN)

#### 2.4.1. Ordinal Encoding for Categorical Items

As shown in Table 2, 10 categorical items which are items no 1 to 10 are encoded to 1 to 30 by ordinal encoding. In this way, it gives categorical data ordinal relationship between them. Categorical data become a continuous data like numerical data.

#### 2.4.2. Embedding

Categorical data are a kind of natural language. Embeddings have been successfully used in Natural Language Processing (NLP). This has the same results in categorical data. It encodes categorical data into a low-dimensional vector representation, which is easily integrable in DLNN.

**Figure 3:**
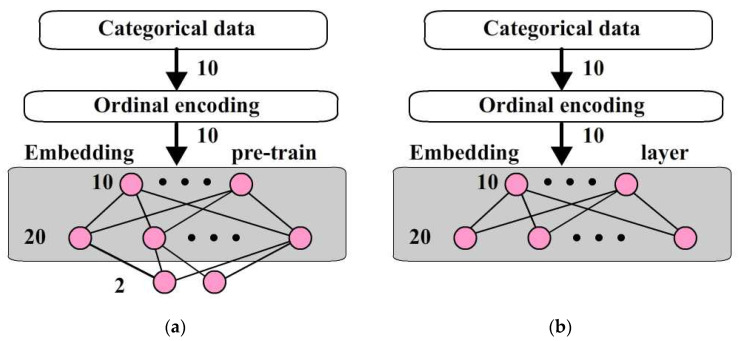
The procedure of generating an embedding layer: (**a**) Pre-train embedding layer; (**b**) Embedding layer.

As shown in Figure 3a, a one hidden layer neural network is used for pre-training with samples to obtain the transform parameters. The output nodes are removed like in Figure 3b to obtain an output vector with 20 nodes. This network is an embedding layer to transform the 10 categorical data into a 20-dimensional vector. In pre-train, 20 is the minimum number of hidden nodes able to achieve a good identification in this case.

#### 2.4.3. Categorical Neural Network

**Figure 4:**
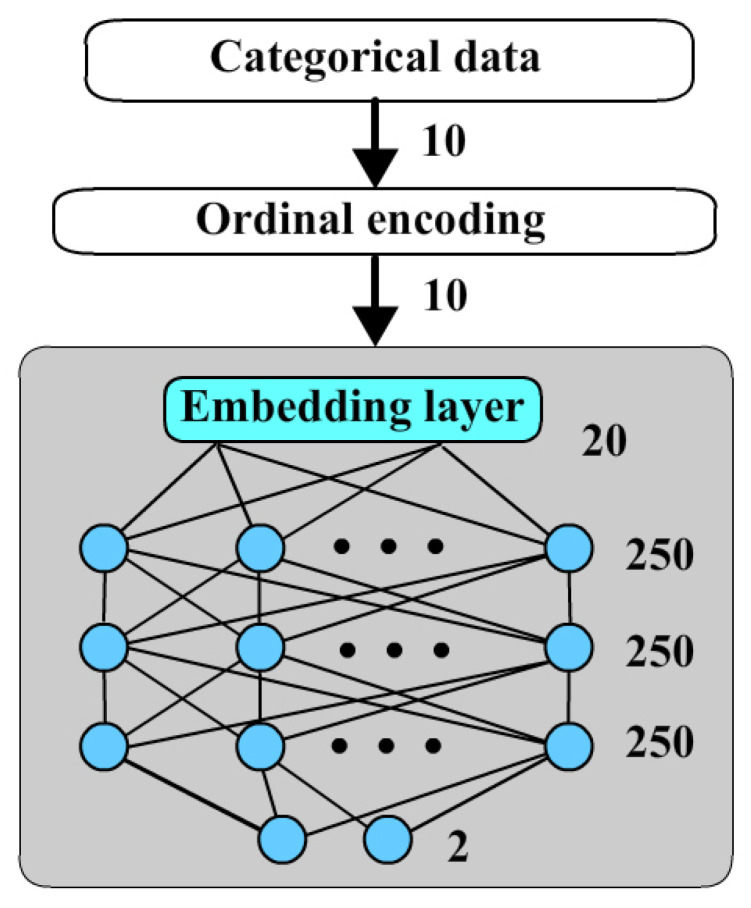
Categorical neural network.

The categorical neural network is structured as Figure 4. Categorical data pass to ordinal encoding and then to neural network. The embedding layer become an input part of this network. It encodes 10 ordinal codes to a 20-dimensional vector.

### 2.5. Three Stage Fusion Neural Networks

**Figure 5:**
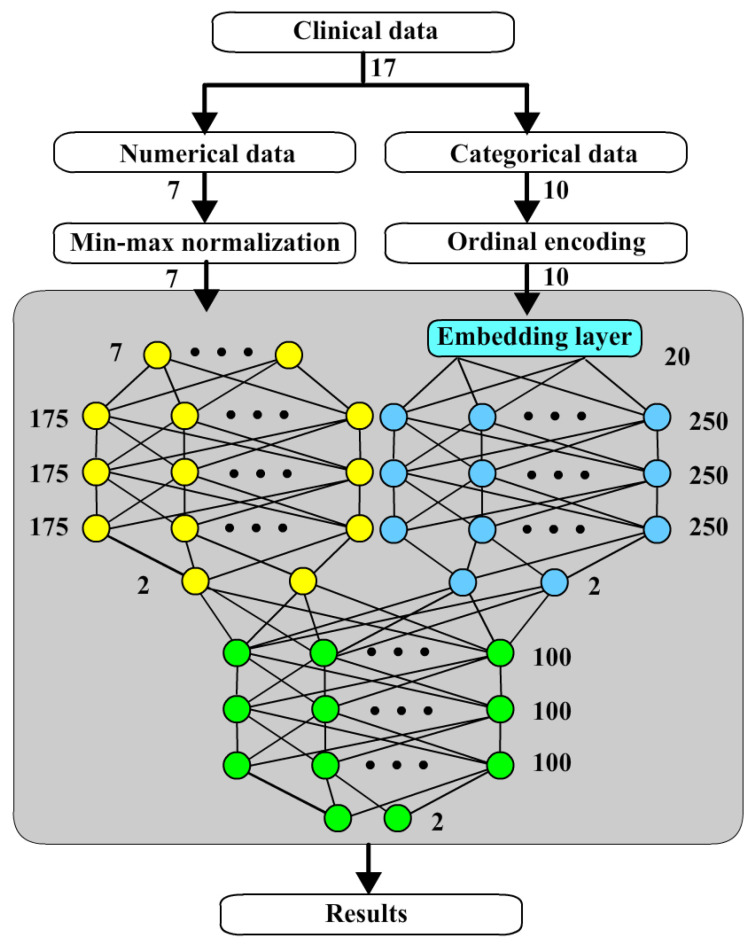
The architecture of TSFNN.

Three-stage fusion neural networks (TSFNN) are made by the combination of NNN and CNN with batch normalization. The architecture of TSFNN is shown in Figure 5. The outputs of NNN and CNN are the inputs of the fusion network.

### 2.6. Batch Normalization

Batch normalization is used to normalize the activations of a layer within a mini-batch during the training of TSFNN. The mean and variance of the activations are calculated for each node within the mini-batch. The activations are normalized by subtracting the mini-batch mean and divided by the mini-batch standard deviation. Parameters are scaled and shifted to the optimal activation distribution. Applying batch normalization in training improves the higher performance of TSFNN.

## 3. Results

The performance of components proposed by our research will be demonstrated in this section. The components include ordinal encoding with embedding layer, data fusion neural network and batch normalization.

### 3.1. Validation Methods

In our experiments, “leave one out cross validation” was used to verify the performance of techniques. It means that one sample is chosen for testing and the other samples are used for training. In this way, 144 samples are used as training samples and one sample is used as a test sample each time. It is run 145 times in total. The results of the following evaluation methods are obtained from the average value of 145 runs.

### 3.2. Evaluation Methods

There are four indexes to evaluate how a TSFNN works. Let fractured teeth be considered positive cases and nonfractured teeth negative cases. True positive number (TP) is the number detecting as positive in positive cases. False negative number (FN) is the number detecting as negative in positive cases. False positive number (FP) is the number detecting as positive in negative cases. True negative number (TN) is the number detecting as negative in negative cases. Accuracy is defined as Equation (2):

Accuracy = (TP + TN)/(TP + FP + TN + FN),(2)

Precision is defined as Equation (3):

Precision = TP/(TP + FP),(3)

Recall is defined as Equation (4):

Recall = TP/(TP + FN),(4)

F1-score is a harmonic mean of precision and recall defined as Equation (5):

F1-score = 2/((1/Precision) + (1/Recall)),(5)

Accuracy shows the detection power for both positive and negative cases; Precision shows the detection accuracy for positivity; Recall shows the detection power in the positive cases; F1-score is an objective result to evaluate total performance.

### 3.3. Performance of Batch Normalization

Table 3 shows the comparison of performance of batch normalization. It is impressive that recall increases the performance from 0.823 to 0.937 about 0.114. However, accuracy achieves half the improvement of recall. This means that batch normalization improves both the positive and negative detection power.

### 3.4. Performance of Embedding Layer

As shown in Table 4, the embedding layer is added to CNN, accuracy and recall gain significant are improved from 0.703 to 0.821 and 0.813 to 0.937 separately. Precision only gains a small improvement. This means that false-negatives are reduced significantly. As a result, this indicates that embedding layer improves true negative detection power for negative cases. It is noted that embedding layer only be applied in processing categorical data. This means that embedding layer is very helpful in processing categorical data.

### 3.5. Performance of Fusion Neural Networks

**Figure 6:**
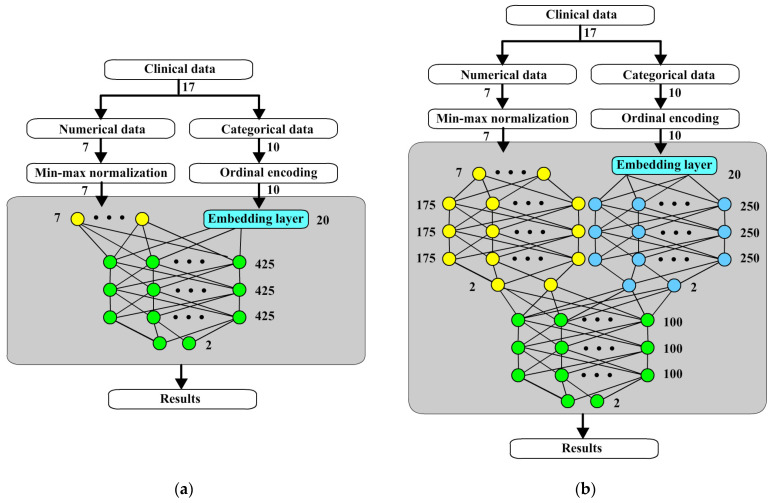
Comparison of two architectures: (**a**) a two-way neural network combination of numerical and categorical data; (**b**) a new architecture of data fusion neural network.

Figure 6 shows two architectures to carry out the comparison of performance. Figure 6a shows a two-way neural network combination of numerical and categorical data without fusion networks. Figure 6b shows the new architecture proposed in this paper. Both of two networks use a similar number of hidden nodes.

Table 5 shows TSFNN could improve two-way NN detection power by about 5%.

## 4. Discussion

This paper builds upon the previous work of Chang [12]. The same variables and same clinical data are used for the research. However, our study proposes a new three-stage data fusion approach and architectures to improve the performance of ANNs. This new approach is not only useful for ANNs but also other NNs. The desired purpose is to understand how to fuse different types of data and neural networks to improve performance. In further research, this architecture will be used to process different medical fields including medical image and signals. Furthermore, “leave one out cross validation” is a good validation method to compare several techniques in the same sample size and variables. Therefore, the experimental results show relative values different for techniques. As the amount of data increases, it is believed the results will also increase proportionally. Additionally, SVM is very sensitive to training data and mixing different types of data. In mixing numerical and categorical data, SVM cannot easily achieve high performance. However, as shown in Table 6, there are different benefits offered by SVM and ANNs. Accuracy and precision of ANNs are better than SVM However, Recall and F1-score of ANNs are worse than SVM. In summary, original ANNs have a similar performance compared to SVM. It is necessary to give more improvement to ANNs to highlight the benefits of neural networks compared to ML in big data. The critical contribution of this study is to show how the performance steadily and gradually improved from ANNs, to two-way ANNs, and then TSFNN.

## 5. Conclusions

Complication of clinical data for diagnosis is very common in medical applications. Medical doctors need to make decisions from a variety of clinical data. These clinical data may include numerical data and categorical data. Even medical image or signal data can be transferred into numerical or categorical data. Fusing such data into a suitable architecture for analysis is a key technique. Sometimes it does not work if data are all entered without carrying out any preprocessing or classifications. It is like “garbage in garbage out”. In such a situation, the accuracy of support vector machine (SVM) and artificial neural networks (ANNs) only achieve 71.7% and 73.1%, respectively.

In this paper, a three-stage fusion neural network is proposed to improve the multiple types of clinical data based on ANNs in the dental field. The target of our research is to predict the risk of root fracture following root canal therapy. Clinical data were obtained from 145 teeth, comprising 97 fractured teeth and 48 nonfractured teeth. Each dataset contained 17 items which describe the dental history and status of patients. They were divided into 10 categorical items and 7 numerical items.

Batch normalization was able to improve NN in F1-score by about 5.5%. Ordinal encoding categorical data with embedding layer could improve the F1-score by 9%. A fusion NN which fuse numerical and categorical NN could improve the F1-score by 4.6%. Combination of all approaches brought an overall improvement of 19.1% in F1-score. This proves three stages fusion neural network is very helpful for handling very complicated data in many medical fields. In all experiments of this study, “leave one out cross validation” was used to verify the performance of techniques. It is fair and trustworthy to compare different models given the limited amount of data. Additionally, it gives a solution that improve the results even in the case of insufficient clinical data.

## Figures and Tables

**Table 1:** A clinical dataset.

No	Items	Options
1	sex	Male, Female
2	previous dental fractures	Yes, No
3	previous prostheses	Yes, No
4	preoperative pain	Yes, No
5	percussion pain	Yes, No
6	endodontical retreatment	Yes, No
7	tooth position	Maxillary anterior teeth
		maxillary molars
		maxillary premolar
		Mandibular front teeth
		Mandibular molars
		Mandibular premolar
8	posts placement	None
		Para post
		Casting post
		Fiber post
		Screw post
9	abutment of removable dentures	None
	or	fixed partial dental prostheses
		Abutment of removable dentures
	fixed partial dental prostheses	Both
10	previous sapicoectomy	None
	or	Previous sapicoectomy
	root amputation	Root amputation
11	the age at the time of treatment	number
12	quantity of remaining tooth walls	number
13	duration from completion of root canal treatment until the date of prosthetic installation	number
14	tooth wear condition	number
15	periodontal condition	number
16	remaining root canal wall thickness	number
17	pericervical dentin thickness	number

**Table 2:** Ordinal encoding code of categorical items.

No	Items	Options	Ordinal Code
1	sex	Male	1
		Female	2
2	previous dental fractures	Yes	3
		No	4
3	previous prostheses	Yes	5
		No	6
4	preoperative pain	Yes	7
		No	8
5	percussion pain	Yes	9
		No	10
6	endodontical retreatment	Yes	11
		No	12
7	tooth position	Maxillary anterior teeth	13
		maxillary molars	14
		maxillary premolar	15
		Mandibular front teeth	16
		Mandibular molars	17
		Mandibular premolar	18
8	posts placement	None	19
		Para post	20
		Casting post	21
		Fiber post	22
		Screw post	23
9	abutment of removable dentures	None	24
	or	fixed partial dental prostheses	25
		Abutment of removable dentures	26
	fixed partial dental prostheses	Both of them	27
10	previous sapicoectomy	None	28
	or	Prvious sapicoectomy	29
	root amputation	Root amputation	30

**Table 3:** Comparison of TSFNN with and without batch normalization.

Architectures	Accuracy	Precision	Recall	F1-Score
TSFNN	0.759	0.818	0.823	0.819
TSFNN with batch normalization	0.821	0.822	0.937	0.874
Improvement	0.062	0.004	0.114	0.055

**Table 4:** Comparison of ordinal encoding with and without embedding layer.

Architectures	Accuracy	Precision	Recall	F1-Score
Ordinal encoding only	0.703	0.757	0.813	0.784
Ordinal encoding with embedding layer	0.821	0.822	0.937	0.874
Improvement	0.118	0.065	0.124	0.090

**Table 5:** Comparison of regular NN and TSFNN.

Architectures	Accuracy	Precision	Recall	F1-Score
Two-way NN	0.752	0.780	0.887	0.828
TSFNN	0.821	0.822	0.937	0.874
Improvement	0.069	0.042	0.05	0.046

**Table 6:** Comparison of SVM, ANNs, two-way ANNs and TSFNN.

Methods	Accuracy	Precision	Recall	F1-Score
SVM	0.717	0.719	0.947	0.817
ANNs	0.731	0.801	0.793	0.796
Two-way ANNs	0.752	0.780	0.887	0.828
TSFNN	0.821	0.822	0.937	0.874

## Data Availability

The data are not publicly available due to privacy or ethical restrictions.

## Abbreviations

The following abbreviations are used in this manuscript:

TSFNN	Three Stages Fusion Neural Network
NNN	Numerical Neural Network
CNN	Categorical Neural Network
FNN	Fusion Neural Network
